# Genetics, Toxicity, and Distribution of Enterohemorrhagic *Escherichia coli* Hemolysin

**DOI:** 10.3390/toxins11090502

**Published:** 2019-08-29

**Authors:** Maike Schwidder, Laura Heinisch, Herbert Schmidt

**Affiliations:** Department of Food Microbiology and Hygiene, Institute of Food Science and Biotechnology, University of Hohenheim, Garbenstr. 28, 70599 Stuttgart, Germany

**Keywords:** *Escherichia coli* hemolysin (EHEC-hemolysin, EHEC-Hly), *Escherichia coli* (EHEC), Shiga toxin-producing *E. coli* (STEC), repeats in toxin (RTX), enterohemolytic phenotype, virulence factor

## Abstract

The ability to produce enterohemolysin is regarded as a potential virulence factor for enterohemorrhagic *Escherichia coli* (EHEC) and is frequently associated with severe human diseases such as hemorrhagic colitis (HC) and the hemolytic uremic syndrome (HUS). The responsible toxin, which has also been termed EHEC-hemolysin (EHEC-Hly, syn. Ehx), belongs to the Repeats in Toxin (RTX)-family of pore-forming cytolysins and is characterized by the formation of incomplete turbid lysis zones on blood agar plates containing defibrinated sheep erythrocytes. Besides the expression of Shiga toxins (Stx) and the locus of enterocyte effacement (LEE), EHEC-Hly is a commonly used marker for the detection of potential pathogenic *E. coli* strains, although its exact role in pathogenesis is not completely understood. Based on the current knowledge of EHEC-Hly, this review describes the influence of various regulator proteins, explains the different mechanisms leading to damage of target cells, discusses the diagnostic role, and gives an insight of the prevalence and genetic evolution of the toxin.

## 1. Introduction

**Figure 1:**
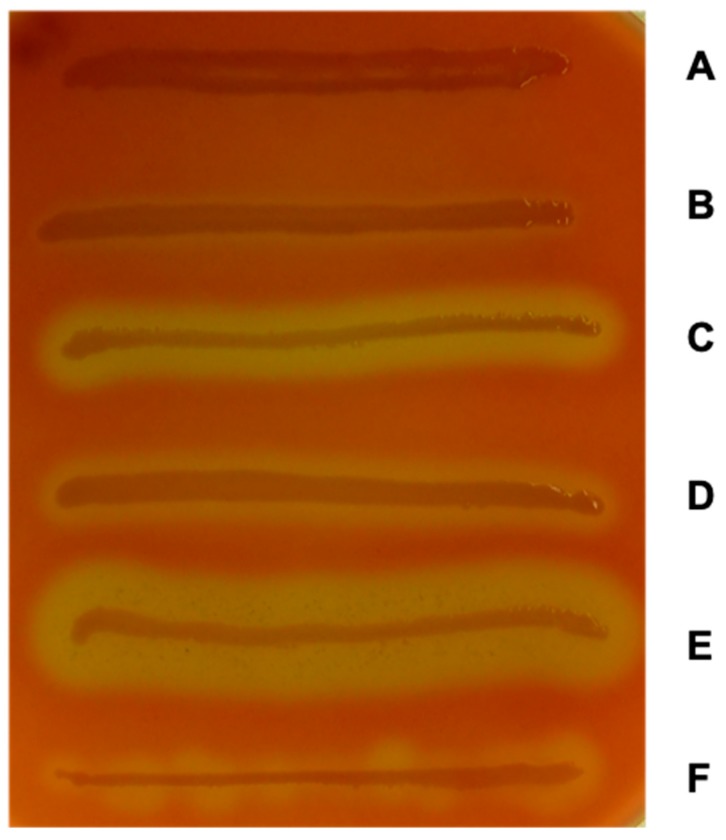
Different hemolysis zones on a blood agar plate containing defibrinated sheep erythrocytes. (**A**) *E. coli* Dh5α is non-hemolytic, (**B**) EHEC O157:H7 strain EDL933 shows the typical enterohemolytic phenotype, (**C**) strain U4-41 shows the typical α-hemolytic phenotype, (**D**) wildtype STEC O113:H21 strain TS18/08 demonstrates the typical enterohemolytic phenotype, (**E**) its isogenic *Δhns* mutant is hyper-hemolytic with large and clear zones of hemolysis, (**F**) and its complemented derivative TS18/08 *Δhns* + *hns* is enterohemolytic. Production of blood agar plates, hemolytic phenotypes, and control strains have been previously described [3,4].

In early studies on the production of hemolysins in pathogenic *Escherichia coli* strains, Beutin et al. described that enteropathogenic *E. coli* demonstrated only a weak hemolytic activity when compared with extraintestinal *E. coli* strains [1]. They could also show that the hemolysin-encoding genes of *E. coli* serotypes O114 and O126 strains were encoded on the chromosome, but those of O26 strains were encoded on a large plasmid [1]. Later on, the authors found this weak hemolytic phenotype frequently in enteropathogenic *E. coli* (EPEC) strains isolated from infants with diarrhea. Therefore, they introduced the term enterohemolysin [2]. The authors characterized the enterohemolytic phenotype by the morphology of the zones of lysis on blood agar plates containing defibrinated sheep erythrocytes. Whereas the typical clear α-hemolytic zone appeared after 3 h of growth at 37 °C, the “enterohemolytic” zones of enteropathogenic *E. coli* O26 strains appeared biphasic after overnight incubation. This was characterized by a clear zone beneath and a small turbid zone around the colony, which looks like an incomplete hemolysis [2]. These studies were extended to Verotoxin-positive *E. coli* strains and Beutin and colleagues found a close association of enterohemolysin and Verotoxin (Shiga toxin)-production [3]. Figure 1 shows the characteristic enterohemolytic phenotype of the enterohemorrhagic *E. coli* (EHEC) O157:H7 strain EDL933 in comparison to the lysis zones of the α-hemolysin-producing UPEC strain U4-41, STEC O113:H21 strain TS18/08 as well as its deletion and complementation mutants, and laboratory strain DH5α which is not able to produce hemolysin. 

Stroeher and Beutin later described enterohemolysin 1 (Ehly1) and Ehly2-DNA sequences which were located on temperate bacteriophages in Shiga toxin-negative *E. coli* O26:H^-^ strains. By molecular techniques, it has been shown that the EHly-1 and Ehly-2 sequences were not present in all hemolytic *E. coli* strains, suggesting the presence of different hemolysins [5]. The frequency of enterohemolytic/enterohemorrhagic *E. coli* strains and the fact that the enterohemolytic phenotype was lost in a plasmid-cured derivative of an *E. coli* O157:H7 strain were the reasons to investigate the nature of the enterohemolysin production in EHEC strains in more detail [6]. In an early study, we could show that the enterohemolytic phenotype of *E. coli* O157:H7 strain EDL 933 was encoded on its large virulence plasmid pO157 and that it was not based on the presence of Ehly-1 and Ehly-2-sequences. Moreover, the enterohemolytic phenotype could be transferred by transformation of plasmid pO157 to laboratory strain *E. coli* HB101. DNA fragments of pO157 were subcloned, respectively, that carried the genetic information for EHEC hemolysin (EHEC-Hly) production [6]. Subsequent studies were then carried out to uncover the genetics of EHEC-Hly production in enterohemolytic *E. coli* strains (see below).

## 2. Genetics of EHEC-Hemolysin

Typical EHEC strains are able to express a number of virulence factors which are encoded on prophages, pathogenicity islands, or large plasmids. Among these, the expression of Shiga toxins (Stx) and the pathogenicity island termed “locus of enterocyte effacement” (LEE) are regarded as the most important virulence factors and are associated with severe humane diseases. Whereas the Shiga toxins, which belong to the class of AB_5_ toxins, exert a cytotoxic effect on Vero cells and HeLa cells [7], the LEE pathogenicity island is essential for the formation of so called “attaching and effacing” (A/E) lesions in the intestinal mucosa. These are characterized by the local loss of microvilli, and an intimate attachment of the bacteria to the enterocytes, together with the formation of pedestal-like structures on the apical membrane [8,9]. In addition, the ability to produce EHEC-Hly is regarded as another potential factor contributing to bacterial virulence [10,11].

As mentioned before, Schmidt et al. have shown that EHEC-*hlyA* (syn. *ehxA*) of EHEC O157:H7 strain EDL933 is located on the large virulence plasmid pO157 [6]. Sequence analysis of pO157 demonstrated two open reading frames with about 60% homology to the *hlyC* and *hlyA* genes of the *E. coli* α-hemolysin (*α-hlyA*) operon [12]. Therefore, they were termed EHEC *hlyA* and EHEC *hlyC* to show the relationship to the α-*hly* genes but also to distinguish them from. Further *in silico* analysis of the predicted amino acid sequence of EHEC-*hlyA* revealed 13 tandemly repeated sequences at the C-terminus as well as the presence of a hydrophobic domain in the amino N-terminal region, which was shown to be involved in pore formation of *E. coli* α-HlyA [13]. Because of these similarities to the *E. coli* α-*hly* operon, the putative EHEC-Hly was also assigned to the RTX-family of pore-forming cytolysins [12]. In a subsequent study, Schmidt and colleagues further analyzed the large plasmid pO157 by sequencing the DNA region downstream of the EHEC-*hlyC* and EHEC-*hlyA* genes [14]. Similar to the *α-hly* operon in *E. coli*, two further genes were discovered and termed EHEC-*hlyB* and EHEC-*hlyD,* respectively. Altogether the four genes are located on the EHEC-*hly* operon with the gene order CABD similar to that of other proteins of the RTX-family [14]. While EHEC-*hlyA* is encoding a 107-kDa protein, which functions as the structural gene for EHEC-Hly, the EHEC-*hlyC* gene product, is assumed to be involved in posttranslational modification of the toxin, similar to α-HlyC of *E. coli* [12,15]. The genes encoding EHEC-HlyB and EHEC-HlyD, in turn, share high sequence similarities with other RTX transport proteins, such as *hlyB* and *hlyD* of *E. coli*, which are required for transport of the toxin out of the bacterial cell [14,15]. 

In a study of Boerlin et al. [16], the diversity of EHEC-*hlyA* in various Shiga toxin-producing *E. coli* (STEC) of different origins, as well as the relationship of EHEC-*hlyA* to the genomic background of STEC was examined. The results obtained by sequencing and Restriction Fragment Length Polymorphism (RFLP) analysis showed that EHEC-*hlyA* is highly conserved among a wide range of STEC serotypes, with approximately less than 4% of nucleic acid substitutions at the maximum [16]. Furthermore, phylogenetic analysis of a subset containing 27 STEC isolates, one enteropathogenic *E. coli* isolate, and a K-12 reference isolate revealed that STEC isolates, positive for the LEE-marker gene *eae*, all evolved out of a single evolutionary lineage. Moreover, the results showed that the large plasmid carrying EHEC-*hlyA* has developed within this lineage, independently of recent horizontal transfer. In addition, two major groups of plasmids (type I and II) carrying the EHEC-*hly* genes were described that were supposed to differed considerably regarding the presence of other virulence-associated factors, as the LEE pathogenicity island seems to be present only in a single phylogenetic lineage harboring the type I plasmid [16]. These findings were later confirmed by studies of Cookson et al. and Newton and colleagues [17,18]. Using PCR and RFLP analysis, Cookson and colleagues performed phylogenetic analysis of EHEC-*hlyA* in *E. coli* strains from different sources (animal and human). According to sequence similarities of the different EHEC-*hlyA* alleles identified, STEC strains could also be divided into two groups, containing *eae*-positive and *eae*-negative strains. Additionally, a third group of *eae*-positive, *stx*-negative *E. coli* (atypical enteropathogenic *E. coli* strains) isolates were analyzed by Cookson et al. (a group which was not included in the previous study of Boerlin et al.), but these strains could all be assigned to the *eae*-positive STEC strains according to their EHEC-*hlyA* sequences. Furthermore, the authors were able to describe six genetically distinct EHEC-*hlyA* subtypes (A to F) [17]. Newton et al. investigated and compared the genetic background and relationship of the large plasmids pO113 of LEE-negative STEC strain O113:H21 and pO157 of LEE-positive STEC O175:H7 strain. By allelic profiling of the EHEC-*hlyA* gene, it was demonstrated that the two plasmids belong to different evolutionary lineages, confirming the results obtained from Boerlin et al., and Cookson et al. mentioned above. Moreover, the virulence plasmids of LEE-negative STEC strains were found to be highly related [18]. 

## 3. Regulation of EHEC-*hlyA* Expression

In *Escherichia coli* O157:H7 strain EDL933, transcription of the EHEC-*hly* operon is influenced by several regulators as it is included in the complex regulation circuit of the LEE pathogenicity island.

**Figure 2:**
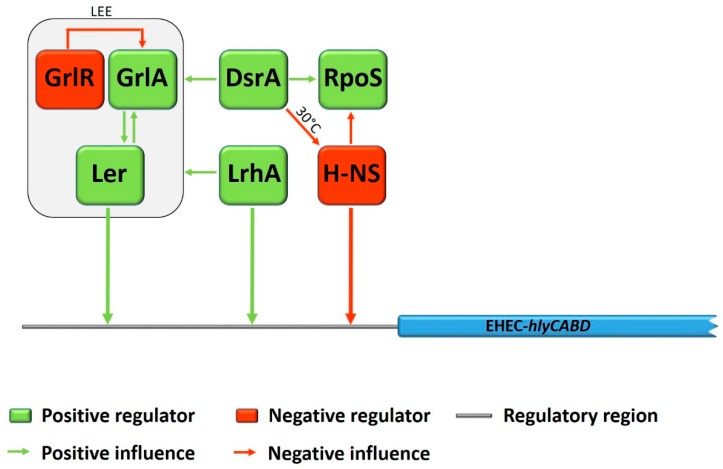
Schematic representation of EHEC-*hlyCABD* regulation. Regulators with positive effect on EHEC-*hly* expression are indicated in green boxes, regulators with negative effect in red boxes. Influences among the different regulators, with regard to EHEC-*hlyCABD* expression, are indicated in green arrows for positive influence or red arrows for negative influence, respectively. Arrows facing the regulatory region of EHEC-*hlyCABD* are indicating a direct influence by binding of the regulator protein (not in scale to the actual binding point).

Three regulators, Ler, GrlR, and GrlA are encoded within the LEE, whereas Ler acts as a global activator for expression of most of the LEE genes [19,20]. In turn, the global regulator of LEE activator GrlA and the global regulator of LEE repressor GrlR are positive and negative regulators, controlling the transcription of the *ler* gene [21]. During a study with deletion mutants in EHEC O157:H7, Saitoh et al. showed that *grlR* deletion significantly increased the lytic activity of EHEC-Hly on plates containing defibrinated sheep erythrocytes, whereas in a *ΔgrlAΔgrlR* double mutant no such effect was observed [22]. Furthermore, the expression of EHEC-*hlyA* was considerably increased by overexpression of GrlA using a multicopy plasmid. This effect could even be demonstrated even in a *ler* deletion mutant, and led the authors conclude that GrlA plays a crucial role in the upregulation of EHEC-*hlyA* genes. Subsequent studies on the regulation of EHEC-*hlyA* gene expression by Iyoda and colleagues demonstrated, that the global LEE regulator Ler also acts as an activator for EHEC-*hlyA* expression in a GrlA-independent way and even more was shown to bind directly to the regulatory region of EHEC-*hlyC* in gel mobility shift assays [23]. In the same study, the non-LEE encoded LysR-type regulator LrhA, which positively regulates the expression of LEE genes [24], was shown to activate the EHEC-*hlyA* expression, even in a *ΔlerΔgrlA* double deletion mutant. Moreover, it was demonstrated that LrhA also activates EHEC-*hlyC* gene expression by direct binding to the regulatory region. See Figure 2 for a schematic overview of influences and effects among different regulators on EHEC-*hly* expression.

During another study with EHEC O157:H7 strain EDL933, Li et al. investigated the influence of the histone-like nucleoid-structuring protein H-NS, the σ factor RpoS, and DsrA on EHEC-*hlyCABD* expression [25]. The small regulatory RNA DsrA is known to inhibit H-NS function and to enhance RpoS synthesis [26,27]. Thus, DsrA was shown to be involved in the regulation of many virulence factors, such as the expression of genes within the LEE [28]. The results of Li et al. indicated that DsrA influences transcription of the EHEC-*hly* operon in a positive manner by two different temperature dependent ways: During cultivation at 30 °C, overexpression of DsrA increased EHEC-*hlyA* transcription in the wild-type EHEC O157:H7 strain, but not in the *hns* deletion mutant. In contrast, at 37 °C, DsrA overexpression stimulated EHEC-*hlyA* transcription in both the wildtype and the *hns* deletion mutant. Therefore, the authors assumed that DsrA might influence transcription of the EHEC-*hlyCABD* operon by a H-NS-dependent way at 30 °C and in an H-NS-independent way at 37 °C. The global transcription regulator H-NS was demonstrated to decrease EHEC-*hlyCABD* expression at 30 °C, whereas no significant influence was shown during growth at 37 °C, and was therefore supposed to be responsible for temperature dependent control of EHEC-*hlyA* expression. The results of this study indicated further that the σ factor RpoS, a known mediator of the general stress response in *E. coli* [29] plays a crucial and primary role in the regulation of EHEC-*hlyCABD* expression, as no levels of EHEC-*hlyA* mRNA could be detected in a *rpoS* deletion mutant, independent of growth temperature or DsrA overexpression. As it was shown before that RpoS concentration was increased in an *hns* deletion mutant [30], the authors assumed that H-NS might influence the expression of EHEC-*hlyCABD* either direct by binding to the upstream regulatory region or indirect by decreasing the expression of RpoS (for an overview see Figure 2). Interestingly, contrary results, regarding the global negative regulator H-NS, were obtained by a study of Rogers et al. [31], in which a deletion of the *hns* gene in STEC O91:H21 strain B2F1 led to a hyper-hemolytic phenotype. Moreover, using electrophoretic mobility shift assays, they could demonstrate that H-NS binds to an 88 bp upstream region of the EHEC-*hly* operon and thus functions as direct negative regulator of EHEC-*hlyCABD* transcription. The authors assumed that the LEE-negative STEC strains used in the study might probably be the reason for the discrepancy to the study of Li et al., who examined the influence of H-NS in a LEE-positive EHEC O157:H7 strain [31]. This assumption is further supported by the results obtained in our group, in which a deletion of *hns* in the *eae*-negative STEC O113:H21 strain TS18/08 (first described in [32]) was constructed for the purpose of another research project concerning subtilase cytotoxin expression [33]. The *hns* deletion mutant showed a hyper-hemolytic phenotype on blood agar plates, whereas the complementation of the mutant with a recombinant *hns* gene on a medium-copy plasmid restored the characteristic wild-type turbid lysis zones. Figure 1 shows the hemolytic phenotypes of different *E. coli* strains. The hyper-hemolytic phenotype of strain TS18/08 *Δhns* can clearly be distinguished from the enterohemolytic phenotype of the wildtype strains. The large clear zone of hemolysis indicates an upregulation of the EHEC-*hly* operon.

## 4. Function of EHEC-Hemolysin and Interaction with Host Cells

Schmidt et al. demonstrated earlier that EHEC-*hlyA* encodes a functional RTX-toxin with a similar pore-forming capacity as the α-Hly of *E. coli* [34]. In experiments with lipid bilayer membranes, EHEC-Hly formed transient ion-permeable channels with a median diameter of 2.6 nm, which is similar to the channel-formation of *E. coli* α-Hly described by Bhakdi and colleagues [35]. Through the insertion of the toxin into the eukaryotic membrane, a change in permeability is caused and resulting in the physical damage of the target cell. Because of the sequence similarities between EHEC-Hly and α-Hly of *E. coli* and the comparable pore size it was suggested that both toxins might function in an identical way. However, differences in the toxic effect on cultured human and bovine were determined. As mentioned before, the studies from Schmidt et al. showed that, EHEC EDL 933 produced very small zones of hemolysis on blood agar plates and showed no hemolytic activity in culture supernatants, which is different to α-Hly of *E. coli* [12]. However, with functioning EHEC-*hlyBD* genes supplied in trans, the hemolysis zones were visible much clearer and a hemolytic activity could be measured in the culture supernatants. Thus, it was assumed that the incomplete lysis might be due to a defective secretion machinery in EHEC strain EDL933. Similar results were obtained by Bauer and Welch [15]. In addition, their results indicated some target cell specificity of EHEC-Hly. Although it did not show an significant lytic effect on two lymphoma cell lines of human origin, complete lysis was observed for BL-3 cells (cells from a bovine lymphoma cell line) and sheep and human erythrocytes [15]. 

Taneike et al. demonstrated that nonpathogenic *E. coli* HB101 induced the production of interleukin-1β (IL-1 β) in human monocytes, after transformation of a recombinant plasmid carrying the EHEC-*hly* operon of an EHEC strain isolated from patient samples [36]. Similar results were obtained in a study of Zhang et al. (2012), in which EHEC-Hly induced the production of IL-1β in human macrophages and was also shown to contribute to cytotoxicity of these cells [37]. As it is known that synthesis of the Stx-receptor Gb_3_ is increased by IL-1β [38], it was speculated, that EHEC-Hly may contribute to bacterial virulence by enhancing the effect of Shiga toxin (Stx) [36]. Additionally, cytotoxicity to human brain microvascular endothelial cells (HBMECs) could be demonstrated for the EHEC-Hly of Stx-negative *E. coli* O26 strains isolated from patients with hemolytic-uremic syndrome (HUS). The authors therefore suggested, that EHEC-Hly might enhance the pathogenicity of Stx-negative *E. coli* O26 strains and may contribute to the development of HUS [39].

It has already been assumed, that EHEC-Hly remains associated mostly to the cell surface, for unclear reasons [12,14]. Aldick and colleagues investigated the grade of EHEC-Hly secreted extracellularly, and found the toxin in two forms: A free, soluble one and one associated to outer membrane vesicles (OMVs) released by EHEC during growth [40], reviewed in [41]. As it is known that many Gram-negative bacteria produce OMVs, which function as vehicle for the transport of toxins and other molecules (reviewed in [42]), Aldick et al. investigated the role of OMVs during EHEC-Hly release. The results of this study showed that EHEC-Hly is associated with OMVs in its biologically active form. Though this association, the toxin was considerably more stable under physiological conditions and showed prolonged hemolytic activity, as compared to free EHEC-Hly. Furthermore, immunogold staining of OMVs containing EHEC-Hly demonstrated that the toxin is located on the exterior surface. For both free and OMV-associated EHEC-Hly, the hemolytic activity was shown to depend strongly on the presence of calcium, a condition which is identical to other members of the RTX toxin family [43]. Additionally, it was demonstrated that the initial binding of OMVs to target erythrocytes is mediated by EHEC-Hly and that this binding step is also calcium-dependent [40]. Since free and soluble EHEC-Hly is known to lyse human microvascular endothelial cells most probably through pore formation in the cell membranes [34,39], the interaction of OMV-associated EHEC-Hly with human microvascular endothelial cells was examined in a subsequent study [44]. By using a model with human brain microvascular endothelial cells (HBMEC) and Caco-2 cells, it was shown that OMV-associated EHEC-Hly did not lyse the target cells via pore formation but triggers apoptotic cell death. The OMV-associated toxin was shown to be first internalized into the target cells via endocytosis and then translocated into endo-lysosomes. Due to endosomal acidification, EHEC-Hly is separated from the OMV and then translocated to mitochondria, resulting in a decrease of the mitochondrial transmembrane potential and cytochrome c release. Through the subsequent activation of caspase-9 and caspase-3, the intrinsic apoptotic pathway is triggered, which leads to death of the host cells [44]. Interestingly, the internalization into host cells via OMVs, and subsequent triggering of the intrinsic apoptotic pathway, was also shown for Shiga toxin 2a (Stx2a) and the cytolethal distending toxin V (CdtV), two major virulence factors of EHEC O157. This indicates the importance of the interactions of EHEC-Hly with OMVs for bacterial pathogenicity [45].

## 5. Detection of EHEC-Hemolysin

During an early study of Beutin et al. [3], various STEC strains isolated from human and animals were analyzed for the presence EHEC-Hly by molecular and microbiological methods. Because of the close association of the production of EHEC-Hly and Stx, EHEC-Hly was proposed as a marker for the rapid detection of potential pathogenic *E. coli* strains [3]. In a subsequent study, Beutin et al. [46] established a system for rapid identification and isolation of STEC from human stool by combining the EHEC-Hly test on blood agar plates with the “reverse passive latex agglutination test for the detection of verocytotoxins (Stx) 1 and 2” (VTEC-RPLA), as a rapid detection system for Stx and EHEC-Hly production. Using this combination, pathogenic STEC strains could be isolated from stool and characterized for toxin production within 72 to 96 h [46]. 

To improve the nonselective enterohemolysin agar described by Beutin et al. [3], Lehmacher and colleagues [47] modified this medium by adding vancomycin, cefixime, and cefsulodin to the blood agar medium (BVCC). Thus, the growth of non-*E. coli* bacteria present in fecal and food samples was inhibited and a competing hemolysis of *Enterobacteriaceae* and Gram-positive bacteria was prevented. After a brief pre-enrichment in mTSB (modified tryptic soy broth) for 6 h and subsequent overnight culturing on BVCC agar, 92.6% of the 95 investigated STEC strains were shown to produce EHEC-Hly. In contrast, only 80% of the same analyzed strains formed hemolysis zones on enterohemolysin agar. Therefore, the authors recommended this method for a reliable isolation and identification of STEC strains from stool and food samples [47].

In a study of Schmidt and Karch [10], a number of 36 STEC O111 strains, isolated from patients suffering from HUS or diarrhea, were characterized for the presence and production of EHEC-Hly. Whereas the enterohemolytic phenotype on blood agar plates was observed in 88% of the samples from patients with HUS, this phenotype could be demonstrated only in 22.2% isolates from patients with diarrhea. Furthermore, two strains showed a nonhemolytic phenotype, although the EHEC-*hlyA* gene was detected by DNA-based methods. Therefore, a combination of Stx-determination and EHEC-Hly characterization was recommended for routine screening of STEC O111 and other non-O157 STEC strains, similar to the results shown by Beutin et al. [46]. The high incidence of EHEC-Hly in isolates from patients with HUS, in contrast to patients suffering from diarrhea, led the authors suggest that EHEC-Hly might increase the capability of the bacteria to cause extraintestinal complications in humans [10].

Different results were obtained in a study of Bai et al., in which stool samples of patients with diarrhea HUS were tested for serogenotypes, *stx* subtypes as well as the presence of the intimin gene *eae* and EHEC *hlyA* [48]. In total, 75 Stx-positive isolates were analyzed, of which 59 isolates carried the EHEC-*hlyA* gene and 35 harbored *eae*. Interestingly, almost all of the *eae* positive isolates, except one, were also positive for EHEC-*hlyA*. The presence of *eae* and EHEC-*hlyA* appeared to be associated with bloody diarrhea, whereas only one of the three HUS isolates carried the EHEC*_hlyA* gene and none of them was positive for *eae*. These results indicate that the correlation of various STEC virulence factors and their involvement in causing clinical symptoms is very complex and remains to be elucidated. Moreover, during analysis of more than 300 STEC strains isolated from patients in Denmark, it was demonstrated that approximately 77% carried EHEC*-hlyA*. However, on the basis of multivariate analysis the EHEC-*hlyA* gene did not play a significant role for the development of severe disease like HUS or bloody diarrhea [49]. 

Although the role of EHEC-Hly in bacterial pathogenicity is still not completely understood, the toxin is meanwhile regarded as a potential virulence factor and commonly used marker for the characterization of STEC strains. For example, EHEC-*hlyA* is one of the genetic markers tested in the U.S. Food and Drug Administration *Escherichia coli* Identification (FDA-ECID) microarray, which provides rapid characterizing of STEC isolates [50]. By using the FDA-ECID microarray, Shridhar et al. [51] analyzed six STEC O104:H7 strains isolated from cattle feces to determine their virulence profiles and to compare the profiles with that of human O104:H7 strains (clinical), the STEC strain O104:H4 (German outbreak strain), and STEC strain O104:H21 (milk-associated Montana outbreak strain). Interestingly, all investigated strains, both bovine and human isolates, were negative for the LEE marker gene *eae*. In contrast, EHEC-*hlyA* was present in four of the six bovine strains, in all human O104:H7 strains, with one exception, and in the Montana outbreak strain (O104:H21). Because all bovine O104:H7 strains were positive for *stx1* and EHEC-*hlyA*, as well as genes coding for various adhesins, this serotype was regarded as a possible foodborne pathogen in humans [51].

## 6. Prevalence and Correlation with Other Virulence Factors

Although EHEC-*hly* genes are found in many STEC serotypes that are associated with diarrheal disease and HUS, the precise role of the respective toxin in the pathogenesis of EHEC is still not completely understood. For example, Gyles at al. [52] investigated the association of the EHEC-*hlyA* gene with different STEC serotypes and found, that EHEC-*hlyA* was more frequently detected in in serotypes which are commonly implicated in disease (89%) than in serotypes that are less frequently involved in disease (46%), indicating an association of EHEC-Hly and pathogenesis of STEC. On the other hand, more than 1000 *E. coli* isolates from healthy cattle were analyzed, showing that the EHEC-*hlyA* gene was only present in 12 independent strains, out of which only 3 strains were also positive for *stx*. Therefore, they concluded that the presence of EHEC-*hlyA* alone is not significant enough for the detection of pathogenic STEC in bovine feces, as the EHEC-*hlyA* gene is present in non-STEC isolates as well [52]. In contrast, Sandhu et al. [53] showed in a subsequent study, using a total of 422 STEC samples isolated from cattle, that 153 samples (~36%) were positive for EHEC-*hlyA* and moreover, that the toxin was present in nearly all of the *eae*-positive isolates (98%) and only in 34% of *eae*-negative isolates. Furthermore, EHEC-*hlyA* was highly associated with serotypes involved in human disease and thus was suggested as a marker for virulence of bovine STEC in humans. However, the authors concluded that EHEC-*hlyA* should not be used to predict the potential virulence of an isolate, as it is also present in STEC which are not implicated in human disease [53].

In a study of Slanec et al. [32] the virulence profiles of various STEC strains, isolated from risk foods such as minced meat or raw milk products, were characterized. The EHEC-*hlyA* gene was one of the most abundant virulence markers detected. Interestingly, EHEC-*hlyA* was found in 30 out of 75 investigated strains (~40%), whereas only 4 strains (~5%) carried the LEE marker gene *eae* [32]. In addition, a number of STEC strains of serotype O113:H21 were isolated during this study, a serotype which is highly correlated with severe diseases in humans [54]. One of these STEC O113:H21 strains, TS18/08, was *eae*-negative, but carried *stx*_2_ and EHEC-*hlyA* as well as the two toxin-encoding genes *subAB* (subtilase cytotoxin) and *cdt-V* (cytolethal distending toxin V) and was therefore be considered as a potential human pathogen.

Similar results, regarding the prevalence of EHEC-*hlyA*, were obtained by Feng and Reddy by analyzing 132 STEC strains isolated from fresh produce [55]. While EHEC-*hlyA* was detected in 61% of the isolates, only about 9% of the isolates were found to harbour the *eae* gene. Similar to the findings mentioned above, these results demonstrate once more that EHEC-*hlyA* is very common among STEC strains found in risk foods [55]. This fact was further confirmed by Boczek et al. [56], during the screening of effluent samples of municipal wastewater treatment plants for the presence of EHEC-*hlyA*. In total, 338 *E. coli* isolates were found to harbor the EHEC-*hlyA* gene, while only 2 isolates were shown to express the *eae* gene. Interestingly, neither *stx_1_*, nor *stx_2_* could be detected in any of the samples. Subsequent serotyping of the isolates revealed serotypes, which were described previously to cause non-O157:H7 EHEC infections in humans, as well as serotypes which have never been reported in association with human infections. These results showed that EHEC-*hlyA* is widely distributed among *E. coli* isolates in the environment but is not automatically an indication for the presence of EHEC [56].

Askari Badouei et al. investigated the occurrence of EHEC-Hly in different diarrheagenic *E coli* strains isolated from cattle and evaluated the association with other virulence markers [57]. Among the 54 EHEC-*hlyA*-positive isolates (~12%), 25 different virulence profiles were observed, reflecting a significant heterogeneity. EHEC-*hlyA* was mostly found in EHEC (72%), but the toxin was also present in enteropathogenic *E. coli* (EPEC) with a percentage of 7.4%. In addition, two potential hybrid EHEC-*hlyA*-positive pathotypes were found consisting of EPEC/ETEC or EHEC/ETEC. Similar to the results described above, these findings indicate that *E. coli* strains isolated from cattle, which are EHEC-*hlyA*-positive, may be genetically considerably heterogenous and may also belong to different pathotypes. 

During a study with more than 400 STEC and non-STEC isolates from animal, food, environmental, and clinical sources, Lorenz et al. [11] investigated the prevalence and subtype patterns of EHEC-*hlyA*. The results obtained by PCR and RFLP revealed 301 EHEC-*hlyA*-positive samples (~69%), as well as six closely related subtypes (A to F), whereby subtypes A and D were found in *eae*-negative STEC and subtypes B, C, E, and F in *eae*-positive STEC strains. Furthermore, EHEC-*hlyA* subtypes differed significantly between isolates obtained from different sources: In animal isolates mostly subtypes A and C were found, in food isolates mainly subtype A was detected, and clinical isolates contained mainly subtype C. Interestingly, only four strains carrying subtype D and two strains carrying subtype E were identified within this study, which were exclusively found in food or clinical isolates, respectively. In addition, this study revealed that certain O serogroups correlated with specific EHEC-*hlyA* subtypes: Subtype A with O104, O113, and O8; subtype B exclusively with O157; and subtype C with O26, O111, and O121 [11]. 

In a later study, Lorenz and colleagues further analyzed the plasmid containing the rare EHEC-*hlyA* subtypes D and E to elucidate the evolutionary relationship among different EHEC-*hlyA* subtypes and to examine their potential role in bacterial virulence [58]. The results showed that the plasmid carrying subtype D was exceptionally large (>200 kbp) and harbored different virulence genes, associated with EHEC as well as with enterotoxigenic *E. coli* (ETEC), although this subtype was only detected in food isolates. The authors therefore assumed that the plasmid carrying subtype D EHEC-*hlyA* represents a novel virulence plasmid, which has evolved from a different evolutionary lineage unlike the plasmids that carry other EHEC-*hlyA* subtypes. In contrast, the subtype E plasmid was smaller in size (50 kbp) and carried only a few virulence determinants even though this subtype was isolated from clinical samples associated with HUS. These findings led the authors suggest that the pathogenicity of this strains might be a result of chromosomally encoded virulence factors, particularly as subtype E isolates were all *eae*-positive STEC strains [58].

In a recent study, Fu et al. [59] investigated the occurrence and genetic diversity of the EHEC-*hlyA* gene in more than 400 non-O157 STEC isolates obtained from human, animal, and food in China, collected in different geographical regions [59]. The results demonstrated that EHEC-*hlyA* was present in 31.8% of the non-O157 STEC strains, a percentage which was lower than that found, for example, in a comparable study of Cookson et al. [17] where EHEC-*hlyA* was found in 63.8% of *E. coli* isolates obtained from cattle and sheep and 96% of isolates from patients with diarrhea. The authors therefore concluded that the prevalence of EHEC-*hlyA* might be different among various geographical locations and sources. Table 1 summarizes the prevalence of EHEC-*hlyA* in different samples origins. Due to sequence analysis of the detected EHEC-*hlyA* genes, Fu et al. [59] determined three different phylogenetic groups which are corresponding to that reported previously by Cookson et al. [17]. Fu and colleagues also demonstrated a clear relationship between EHEC-*hlyA* groups and their sources, similar to the findings of Lorenz et al. [11].

## 7. Conclusions

Although the precise role of EHEC-Hly in the pathogenesis of EHEC and other virulent *E. coli* strains is yet not fully understood, the toxin is recognized as a potential virulence factor and a commonly used virulence marker for the analysis of EHEC and STEC strains. The EHEC-*hlyA* gene is found very frequently in STEC isolates which belong to serotypes associated with severe human diseases. However, the clinical importance of this toxin alone is still unclear, as the association with either HC or HUS cannot be clearly assigned. This indicates that the clinical effect of EHEC-Hly might be very complex and depends on the correlation with several other virulence factors. Furthermore, EHEC-Hly is also present in environmental *E. coli* isolates, which are lacking Stx and thus should only be used in combination with other virulence factors for an estimation of the bacterial virulence potential. Further studies are necessary to investigate the role of EHEC-Hly in combination with Stx and other virulence factors for the development of EHEC-associated diseases. This could be achieved in several models using modern mutagenesis techniques such as CRISPR/Cas in combination with appropriate cell cultures and organoid models. Although the mode of pore-formation has been elucidated, more in depth research should be conducted on the mode of action and target structures. Moreover, the interaction with global regulators and their influence on EHEC-*hlyA* expression should be investigated further to elucidate the clinical importance of EHEC-Hly. In addition, the characterization of different EHEC-*hlyA*-subtypes could be a method for surveillance and documentation of EHEC infections, as a clear association between different subtypes and sample origins, as well as O serotypes, was shown.

## Figures and Tables

**Table 1:** Prevalence of EHEC-*hlyA* obtained from different sources.

Sample Origin	Percentage of EHEC-*hlyA* Positive Samples	Reference
*E. coli* isolates from normal cattle	<1	[52]
STEC isolates from normal cattle	36%	[53]
STEC isolates from risk foods	40%	[32]
STEC isolates from fresh produce	61%	[55]
*E. coli* isolates from effluent samples	338 positive samples *	[56]
Diarrheagenic *E. coli* strains from cattle	12%	[57]
STEC and non-STEC isolates from human, animal, and food	69%	[11]
non-O157 STEC isolates from human, animal, and food	31.8%	[59]
*E. coli* isolates obtained from cattle and sheep	63.8%	[17]
STEC isolates from patients with diarrhea.	96%	[17]

* total no. of samples was not described in [56].
